# Exploring why global health needs are unmet by research efforts: the potential influences of geography, industry and publication incentives

**DOI:** 10.1186/s12961-020-00560-6

**Published:** 2020-05-15

**Authors:** Alfredo Yegros-Yegros, Wouter van de Klippe, Maria Francisca Abad-Garcia, Ismael Rafols

**Affiliations:** 1 Centre for Science and Technology Studies (CWTS), Leiden University, Leiden, The Netherlands; 2 Department of History of Science and Documentation, Universitat de València, València, Spain; 3 Ingenio (CSIC-UPV), Universitat Politècnica de València, València, Spain

**Keywords:** burden of disease, unmet health needs, pharmaceutical industry, research priorities, research evaluation, publication incentives

## Abstract

**Background:**

It has been well established that research is not addressing health needs in a balanced way — much more research is conducted on diseases with more burden in high-income countries than on those with more burden in lower-income countries. In this study, we explore whether these imbalances persist and inquire about the possible influence of three factors, namely geography, industry and publication incentives.

**Methods:**

We use WHO data on the Global Burden of Disease as a proxy measure of health needs and bibliometric information as a proxy for research efforts. Scientific publications on diseases were collected from MEDLINE using MeSH terms to identify relevant publications. We used Web of Science to collect author affiliations and citation data. We developed a correspondence table between WHO ICD-10 and MeSH descriptors to compare global health needs and research efforts. This correspondence table is available as supplementary material.

**Results:**

Research output is heavily concentrated in high-income countries and is mainly focused on their health needs, resulting in a relative lack of attention to diseases in lower income countries. A new finding is that diseases with a similar burden in high- and middle-income countries are also under-researched, both globally and in relation to disease burden in high- and middle-income countries. Global industrial R&D is found to be very similar to the focus of public research. Diseases more prevalent in high-income countries generate ten-fold more research attention than those in low-income countries. We find no discernible preference towards diseases of high-income countries versus those of low-income countries in the top 25% most prestigious journals. However, in middle-income countries, citation rates are substantially lower for diseases most prevalent in low- and middle-income countries.

**Conclusions:**

From a global perspective, the imbalance between research needs and research efforts persists as most of the research effort concentrates on diseases affecting high-income countries. Both pharmaceutical companies and the public sector also tend to focus on diseases with more burden in high-income countries. Our findings indicate that researchers in middle-income countries receive more citations when researching diseases more prevalent in high-income countries, and this may divert the attention of researchers in these countries from diseases more prevalent in their contexts.

## Background

The discussion of the 10/90 gap of biomedical research, which highlights that “*only about 10 percent of the global biomedical research budget is allocated to diseases accounting for about 90 percent of the world’s health problems*”, was first introduced in a report 20 years ago [1, 2]. While this estimate may be out of date, and its accuracy disputed [3], the general observation is still relevant. The lack of balance between disease burden and global health research attention has been well-documented by different studies using WHO Global Burden of Disease data, on the one hand, and different estimates of research effort, such as project funding or publication data, on the other [4–10]. In order to have more robust information on research and development (R&D) investment on neglected and poverty-related diseases, the G-Finder initiative and WHO Global R&D Observatory were recently set up [9] (https://gfinder.policycuresresearch.org/and https://www.who.int/research-observatory/en/).

Despite broad recognition of the scale of this misalignment and a variety of funding initiatives, major imbalances in relative research investment persist [11]. A larger shift in research priorities is required and, to achieve it, greater understanding of the factors contributing to this imbalance is necessary. In this paper, we set out to explore some of the reasons why research fails to address some global health needs. In order to capture patterns that facilitate broad public and policy discussion, we carry out an analysis in terms of the four income levels of countries and five disease types [9]. These coarse classifications are helpful in capturing the main trends of disease burden and research attention; however, they should be complemented with more fine-grained studies, for example, for specific countries [12, 13] and diseases [14].

Although our interest is on research, it is important to bear in mind that perceptions of health needs and disease burdens are affected by a variety of local factors such as healthcare systems, environmental and living conditions, cultural preferences, social inequality and access to education. In some cases, e.g. in maternal complications, there are medical solutions that are not implemented or not accessible for part of the population and more biomedical research is not the solution. Therefore, research should not solely be driven by disease burden, nor should reducing disease burden be thought of as solely contingent upon the availability of biomedical research. A one-to-one relation between research efforts and burden should not be expected.^1^

Yet, research efforts are important to address disease burden in two respects. First, to produce new knowledge that brings about improvements in the prevention, diagnosis, treatment or management of diseases, i.e. to generate new solutions, but also, and possibly more importantly, to create capabilities in human resources and infrastructure for innovation to take place, generally outside of academia, in hospitals, governments or companies. Innovation studies literature has long claimed that the main contribution of research is to create capabilities rather than off-the-shelf solutions [15, 16].

We investigate these driving factors using WHO data from Global Health Estimates on Disability-Adjusted Life Years (DALYs) for disease burden and Web of Science publication data as a proxy of amount of research efforts. We view health and R&D indicators such as those presented here as proxies that can be questioned and contested. Nevertheless, we believe that they provide information that can be useful for opening public debates and suggesting avenues for further research [17, 18].

This paper makes three contributions. First, it provides an updated comparison of publications against disease burden that results in new findings regarding the relative lack of investment in diseases with high burden in middle-income countries. Second, it explores the driving factors for the imbalance between research efforts and global disease burden, including new bibliometric data regarding pharmaceutical research agendas and citation incentives. Finally, it makes publicly available a correspondence table between the definitions of the WHO International Classification of Disease (ICD-10) and the disease descriptor Medical Subject Headings (MeSH) of the United States National Library of Medicine.

Establishing the factors influencing research agendas is extremely difficult because they are shaped by many interacting social, economic and political factors [19–22]. As Gläser and Laudel highlight, “*researchers need to select problems and approaches that create a sufficient agreement between their own interests, the* [scientific] *community’s expectations concerning relevant and reliable contributions, and expectations by external actors*” ([21], p. 436). In this study, we limit the exploration to three potential factors – research prioritisation driven by national (rather than global) health needs; the influence of pharmaceutical companies; and incentives to publish in prestigious journals and on highly cited topics. There are other factors influencing the choice of disease topic that we do not explore here; most importantly, the expectation that progress can be achieved by doing research in a certain field – whether research is needed and whether the problems are practically ‘doable’ [23].

Given that most public research is funded and governed at the national level, it should be of no surprise that research priorities are more balanced in relation to national health needs than to global health needs. A higher correlation between health needs and research at the national level is indeed observed [5]. However, since knowledge production is heavily concentrated in high-income countries (HICs) [24] but overall disease burden is higher in low- and middle-income countries (LMICs), one can expect a great lack of balance between global research and global health needs. One can nevertheless wonder whether priorities in LMICs are driven by their own needs.

A second prominent factor is market demand. According to Røttingen et al. [9], private companies make up around 60% of the total health R&D, with pharmaceutical companies leading this investment. For example, companies such as Roche spend around 9bn Euro per year in R&D (https://ec.europa.eu/info/news/2018-industrial-rd-scoreboard-eu-companies-increase-research-investment-amidst-global-technological-race-2018-dec-17_en), which is about seven times the budget of a major funder of biomedical research such as the Wellcome Trust. Prior research has indicated that the majority of market demand for health research is found within chronic conditions predominantly affecting individuals in HICs. As a result, research funded by pharmaceutical companies most often investigates these diseases [25–27]. This funding from industry is likely to indirectly influence the topic selection of researchers, even when the studies are funded with public money. In other words, pharmaceutical research priorities are likely to ‘spillover’ into public research priorities. Indeed, Evans et al. [5] found that the global health research efforts were positively related to market for treatment but negatively related to global disease burden.

A third factor influencing research agendas is the academic prestige and evaluation pressure associated with norms and incentives within scientific institutions. This influence may be indirect and informal, through the selection of topics that are seen as prestigious within a certain scientific community, and are thus likely to contribute positively to researchers’ career developments. Alternatively, it can also be fully institutionalised in national evaluation systems that reward scientific visibility, often proxied through either the prestige of the journals that the authors publish in (generally via the Journal Impact Factor (JIF)) or the number of citations their publications receive [28].

These forms of assessment may be harmful when used without care [29, 30] and, in some cases, may incentivise researchers in ‘peripheral’ countries (not only in LMICs, but also in some ‘peripheral’ HICs like Italy or Spain) to conduct research that is relevant for large HICs rather than focusing on local or national needs [31–34]. In the case of health, the hypothesis is that diseases more prevalent in HICs are more likely to be published in prestigious journals (e.g. with higher JIF), get more citation attention, and may thus be more attractive for the purpose of career advancement and academic recognition.

## Methods

### Disease burden estimates

We used the Years of Life Lost and DALYs provided by WHO in 2017 as a proxy of the burden of disease [35]. Although DALYs are not free of limitations, they are one of the most established proxies of disease burden [36–38]. We used the DALY estimates for 2010 in order to analyse (via publications) whether research responded to health needs in 2010 in the following years (2010–2014). Additionally, choosing this time segment allowed for a detailed analysis of the citation impact of the publications published between 2010 and 2014. Our analysis is based on the 134 specific diseases within the groups of diseases (1) communicable, maternal, perinatal and nutritional conditions and (2) non-communicable diseases (Additional file 3).

### Bibliometric methods for estimating research efforts on disease

**Fig. 1:**
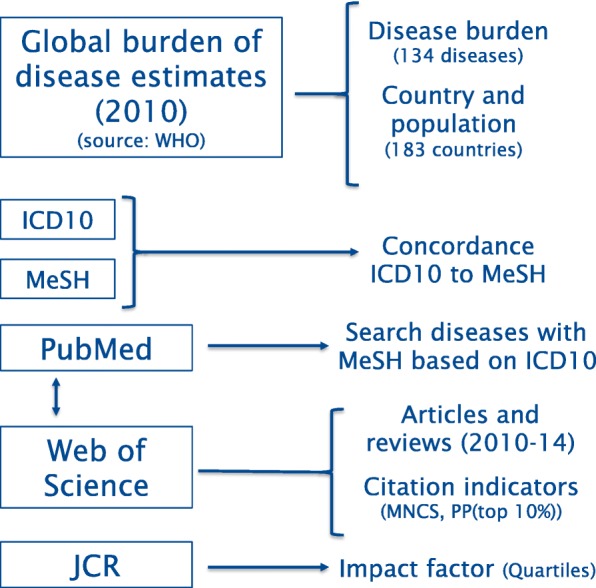
Summary of the data collection strategy of disease burden and publication data

We relied on scientific publications as a proxy of the research effort made on different diseases for the period 2010 to 2014. These publications were collected from MEDLINE in May 2019 through the MeSH terms that best describe the 134 diseases considered in the analysis. We built a concordance table between ICD-10 (classification used by WHO) [35] and MeSH terms. Despite some serious limitations, we expect the data to capture general trends and dynamics reflecting some aspects of the research conducted on these diseases. The Centre for Science and Technology Studies (CWTS) in-house version of the Web of Science was used to retrieve additional information from publications such as the institutional affiliations of the authors and the number of citations received by these publications. We limited the analysis to articles and reviews. Figure 1 provides a summary of data collection methods.

### Classification of countries by income groups

Publications are attributed to countries on the basis of the authors’ country of affiliation using fractional counting. We grouped countries into four categories, according to their income level in the year 2010, following the World Bank’s historical classification of countries by income (https://datahelpdesk.worldbank.org/knowledgebase/articles/906519-world-bank-country-and-lending-groups) as HICs, upper–middle-income countries (UMICs), lower–middle-income countries (LoMICs) and low-income countries (LICs). It is important to keep in mind that certain countries contribute to a large proportion of the overall scientific production of their income level group. For example, the United States represents 35% of publications of HICs, China produced half of the publications in the UMICs group and India produced 60% of all publications in LoMICs.

### Classification of diseases by relative burden over HICs or LMICs

We grouped the 134 diseases considered in the analysis into five disease types according to their relative burden in HICs and LICs. This categorisation is based on a modified version of the WHO framework developed in 2012 [9, 39]. The initial categorisation classifies diseases as type 1, 2 and 3, depending on whether the burden generated by the diseases is relatively larger in HICs or in LMICs. Given that type 1 was very large, we further split the group of diseases into type 1a (more burden per capita in HICs), type 1b (equal burden in HICs and LMICs) and type 1c (just a bit more burden in LMICs), inspired by the work by von Philipsborn et al. [10], as described in Table 1. The assignment of specific diseases to types can be found in supplementary Additional file 2.

**Table 1:** Classification of diseases according to differences of burden per capita in low- and middle-income countries (LMICs) compared to high-income countries (HICs)

Type	Relative disease burden per capita^**a**^	Description	# Diseases	Exemplary cases
**1a**	< 0.75	More burden in HICs	34	Colon cancer, breast cancer, Alzheimer’s disease
**1b**	0.75 ≤ x < 1.25	Equal burden	28	Depression, schizophrenia, ischemic heart disease
**1c**	1.25 ≤ x < 3.00	A bit more burden in LMICs	26	Cirrhosis, stroke
**2**	3.00 ≤ x < 35.0	More burden in LMICs	22	Maternal conditions, HIV
**3**	≥ 35.0	Quasi exclusive of LMICs	24	Malaria, diarrhoeal diseases

^a^Relative disease burden per capita is calculated as the ratio of disease burden per capita in LMICs over disease burden per capita in HICs [10]

### Journal impact factor analysis

We collected the JIF of the journals where the articles included in our analysis where published. This information was extracted from the Clarivate Analytics’s Journal Citation Reports. The impact factor (IF) reflects the average frequency with which articles published in a given journal are cited in a given period of time. Based on all IFs, we were able to determine the quartile to which the journal belongs. We followed the same procedure as in the Journal Citation Reports (http://help.incites.clarivate.com/inCites2Live/indicatorsGroup/aboutHandbook/usingCitationIndicatorsWisely/jifQuartile.html) to obtain these quartiles, so that quartile Q1 groups the 25% of journals with the highest IFs within a given subject category.

### Citation analysis

We have computed two citation-based indicators as proxies of the scientific impact of publications. The first indicator is the Mean Normalised Citation Score, which reflects the average citation impact of a set of publications. The second measure indicates the proportion of publications produced by a given aggregate that belong to the top 10% most frequently cited worldwide. Both indicators are normalised by scientific field and publication year. This means that the publications we included in our analysis are compared to publications published worldwide the same year and in the same scientific field [40, 41].

Further details on data and methods can be found in the supplementary Additional file 1. The data of the figures, including some complementary information, is available in Additional file 2. The concordance table is available in Additional file 3.

### Research collaboration

Several studies have systematically found a strong relationship between research conducted in collaboration, especially international collaborations, and citations [42, 43]. In order to account for the possible influence of collaboration in analyses of citation impact, we have distinguished between publications with and without international collaboration. We considered publications listing two or more different countries in the affiliations of the authors to be produced in international collaboration.

### Publications produced and funded by the pharmaceutical industry

We have considered 23 large pharmaceutical companies in order to analyse the priorities of big pharma across disease types. Thus, instead of considering an exhaustive list of companies active in the pharma sector, we focus on the largest companies in the sector, which are those funding more research to academia.

We have identified two groups of publications related to big pharma – (1) publications (co-)authored by researchers working at these companies and (2) publications that acknowledge that the research has been conducted with financial support from these companies. Further details on the identification and process of this information is provided in the supplementary materials (section S1.3) and the website www.cwts.nl/bigpharma.

## Results

### Comparison of global disease burden versus global research efforts

**Fig. 2:**
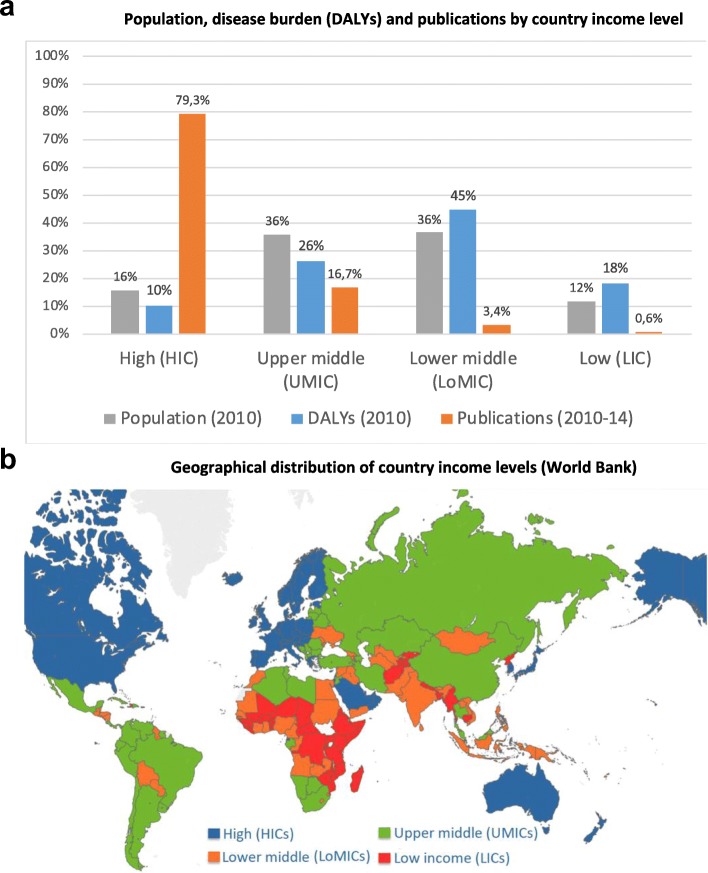
**a** Population, disease burden (DALYs) and publications by country income level. **b** Geographical distribution of country income levels (World Bank)

Figure 2a shows that HICs produce a disproportionately large number of publications (close to 80%) compared to the percentage of the global population living in these countries (16%) and the disease burden affecting them (10%). On the contrary, LMICs make up roughly 85% of the global population and 90% of the global disease burden, while they only produce around 20% of publications. It is worth highlighting as well that UMICs produce most of the research outside HICs (17% of total). UMICs include China, most of Latin America, Russia and South Africa (Fig. 2b). LoMICs, whose production is dominated by India, account for 3.4% of publications. LICs only produce 0.6% of the world’s publications.

**Fig. 3:**
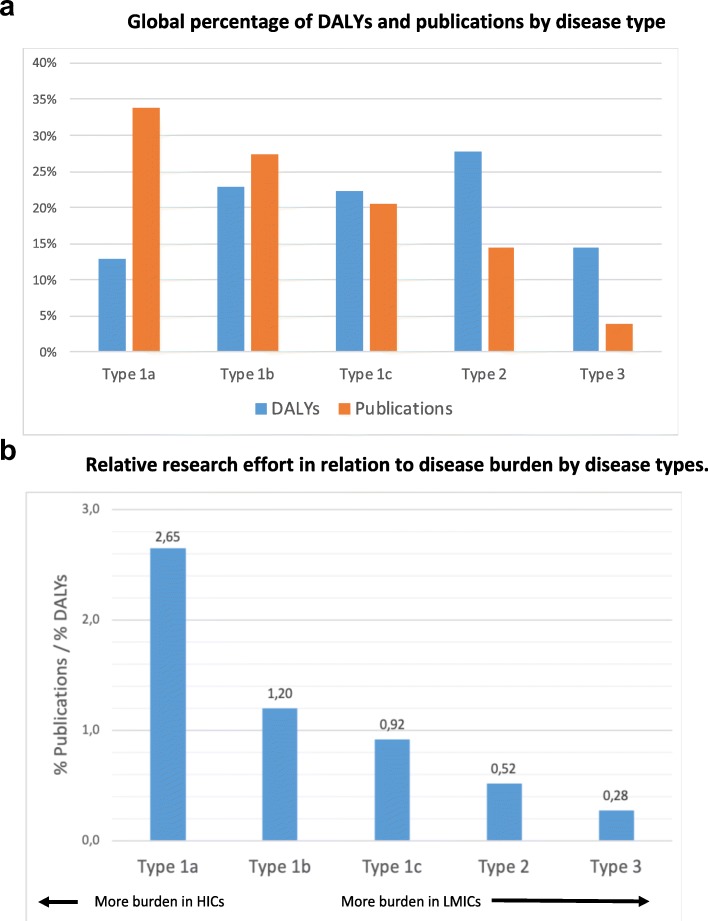
**a** Global percentage of DALYs and publications by disease type. **b** Relative research effort in relation to disease burden by disease types

Figure 3a shows the relative (%) disease burden against the relative (%) of publications for various disease types (listed in Table 1 below). We observe that, although global disease burden is quite spread across disease types, the percentage of publications declines steadily from type 1a (similar prevalence in HICs and in LMICs) to type 3 (more prevalent in LMICs than in HICs). Type 1a account for the lowest percentage of disease burden worldwide (13%) but the largest share (34%) of scientific publications. In contrast, type 3 diseases make up only 4% of the world’s publications while contributing to 14% of the global disease burden.

Figure 3b shows the ratio of the relative research effort over the relative disease burden. This ratio is an estimate of investment per unit of disease burden (% of publications over % of DALYs for a disease type). Type 1a diseases have ten times more publications per DALY than type 3, five times more than type 2, and two times more than type 1b. The figure suggests that R&D investments have a strong systematic imbalance against the diseases mainly or only affecting LMICs.

### The geographic distribution of disease burden and research efforts

Given that the relative burden of diseases are unequal across geographical regions and countries of disparate income level, it is to be expected that research efforts also follow national patterns [5].

**Fig. 4:**
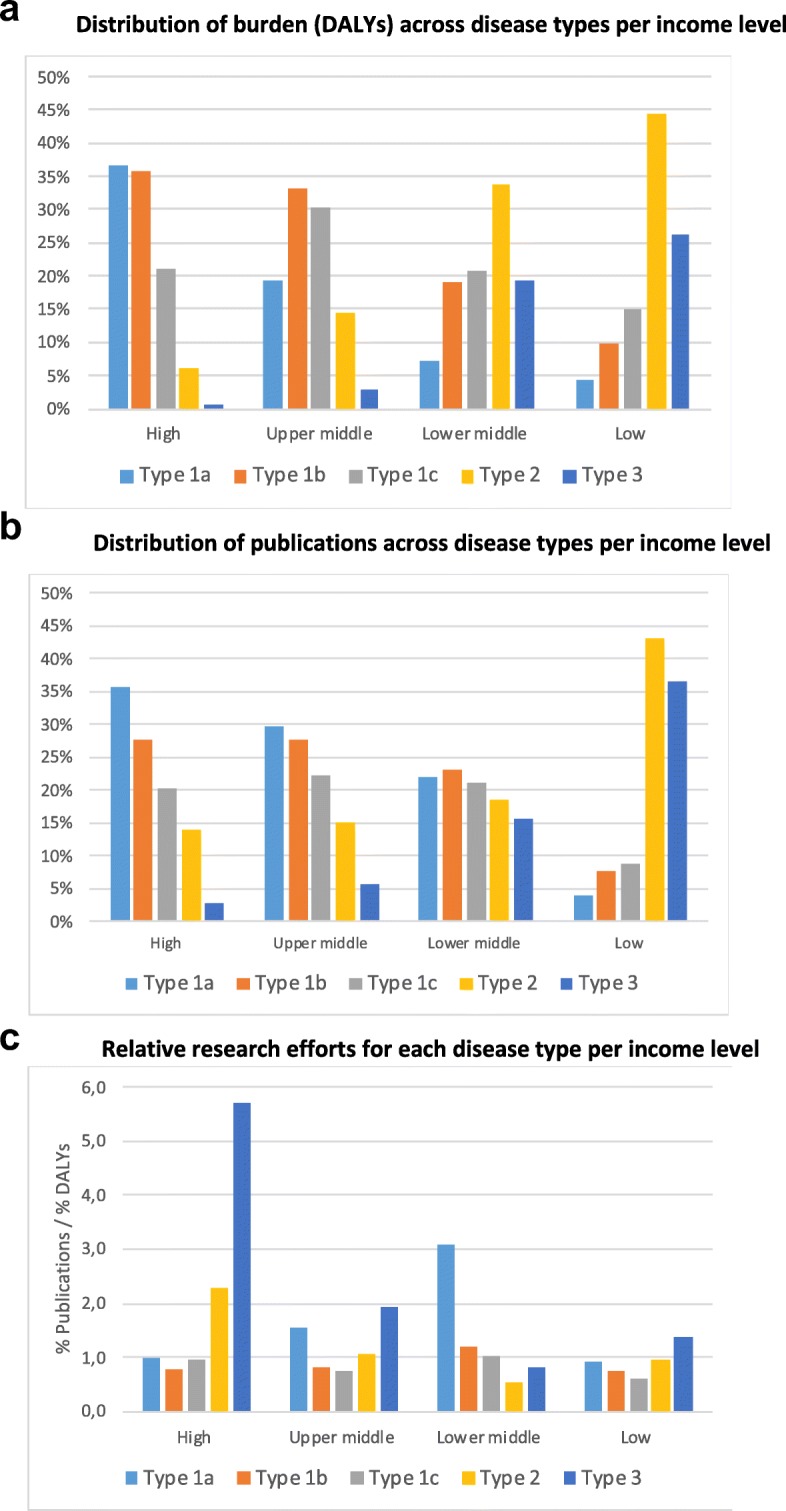
**a** Distribution of burden (DALYs) across disease types per income level. **b** Distribution of publications across disease types per income level. **c** Relative research efforts for each disease type per income level

Figure 4a shows the distribution of DALYs across disease types for different income levels. We observe that type 1a and 1b make up more than 70% of the burden in HICs, whereas types 2 and 3 account for more than 70% of the burden in LICs. MICs show transition patterns, with their highest burden shifting from type 1b and type 1c (high in UMICs) to type 2 (high in LoMICs).

Publication distributions show similar patterns across income country level, as illustrated in Fig. 4b. In HICs, publication shares by disease type decrease steadily from type 1a to type 3, whereas in LICs, we observe that both type 2 and type 3 diseases constitute research priorities, with almost 70% of the publications. These results are thus in agreement with Confraria’s [44] findings that African health research focuses on their pressing health needs and that international collaboration and funding supports this focus. Nevertheless, the number of publications by HICs is so much larger than that of LMICs that the 3% of HIC publications devoted to type 3 diseases amounts to more than half (58%) of all publications worldwide devoted to type 3 diseases, while the 36% of LIC publications on type 3 amounts to only 6% of the global production (shown in Fig. S3, Additional file 2).

It should be noted that there are some interesting departures from ‘alignment’ (better observed in Fig. 4) that show the ratio of the % of publications over the % of DALYs. First, we observe that HICs conduct much more research on types 2 and 3 (six-fold and two-fold, respectively) than could be expected from their own disease burden. Such research capacity in HICs for diseases occurring almost exclusively in LICs can be explained by HIC’s colonial legacies and programmes of international cooperation [11]. UMICs also conduct more research on type 3 diseases than would be expected (two-fold). This is understandable since countries like Brazil are potentially vulnerable to and have a historical expertise in tropical and neglected infectious diseases.

More unexpectedly, though, is the observation that MICs conduct more research than would be expected from their observed disease burden on the diseases that are most relevant to HICs. In UMICs (with China as main contributor), type 1a diseases account for 30% of publications, although their burden is only 19%. However, types 1b and 1c, which have the largest burden (more than 60%) in UMICs, make less than 50% of publications. Similarly, in LoMICs (which is dominated by India’s production), type 1a diseases get a three-fold attention above expectation, whereas type 2 (which is by far the largest, with 34% of DALYs) and type 3 are relatively under-researched. These imbalances point to potential problems in the priorities of UMICs and LoMICs and deserve further analysis.

### The focus of pharmaceutical research efforts

Most details on big pharma’s research priorities are not public. However, it is possible to estimate their relative research efforts through their own publications, and the publications that acknowledge their funding, which are mainly by universities doing contract research for them. Although the number of these publications is relatively small (of the order of 12,000 published and 12,000 funded per year against 600,000 articles and reviews published on health areas per year), these companies do conduct considerable amounts of R&D. In fact, it is estimated that business expenditure makes up about 60% of global health R&D [9]. Companies such as Roche have budgets of the order of 10bn US$, whereas government R&D expenditure in health is of the order of 1–2bn US$ in countries like the United Kingdom, France or Germany. Thus, the large scale of pharma R&D expenditure suggests that it is likely to influence researchers’ priorities, even if their number of publications are modest.

**Fig. 5:**
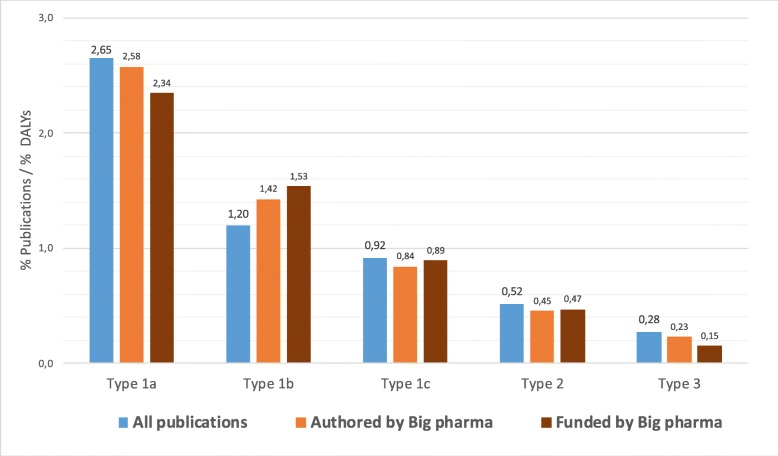
Relative research effort by disease type by ‘big pharma’

Here, we provide data on the research focus of Fig. 5 and show the relative research efforts of big pharma by disease type that were published (orange) and funded (brown) by 23 pharmaceutical companies. A list of publication trends and specialisation patterns by specific diseases of these 23 companies is available at http://www.cwts.nl/bigpharma. Since big pharma companies may be expected to follow market demands rather than health needs, it is not unexpected that they publish and fund more research in type 1a diseases than in other types. Rather surprisingly is the extent to which their publication and funding patterns reflect those of all publications on diseases, which are mainly conducted by public research organisations.

One would expect public research to focus on topics other than those focused on by private research. Indeed, one can make the ‘market failure’ argument, namely that public research priorities should be set in a way to fill gaps in knowledge that would otherwise receive relative under investment if left to market mechanisms. However, the similarity in priorities observed suggests that public and private biomedical research are strongly coupled. It seems plausible that big pharma shapes research priorities by funding part of the research in public labs as well as influencing a variety of decision-making processes in the public domain [45].

### Journal prestige and citation patterns across disease types

Academic reputation is another factor that may be influencing research topic selection. It is often argued that research topics not relevant for HICs are less likely to be accepted in prestigious journals and attract attention (and citations!) [31, 34]. We have examined these hypotheses in the ensuing analyses.

**Fig. 6:**
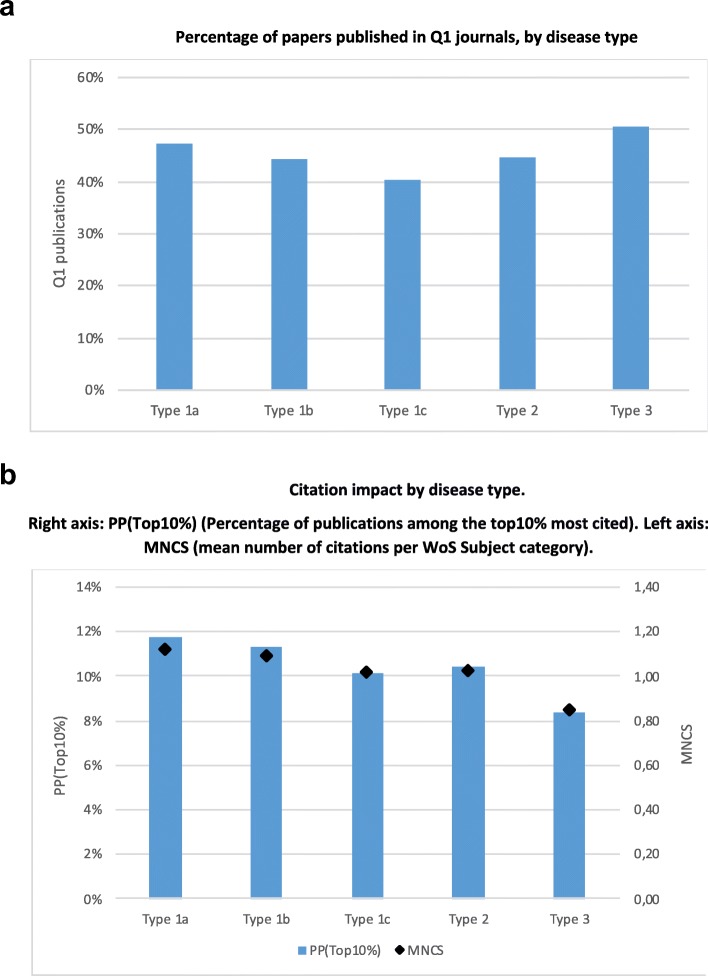
**a** Percentage of papers published in Q1 journals, by disease type. **b** Citation impact by disease type. Right axis: *PPTop10%* Percentage of publications among the top 10% most cited. Left axis: *MNCS* mean number of citations per Web of Science Subject category

Figure 6a shows the percentage of publications in the top quartile of JIF (Q1)^2^ across disease types. This choice of Q1 follows a conventional ‘quality’ criterion in formal evaluation practices [28]. The choice of Q1 is highly problematic as an indicator of ‘quality’ [30], but it may represent the pressure experienced by researchers via both formal and informal evaluation regimes. The pattern observed does not support the hypothesis that diseases with more burden in LMICs are less likely to be accepted in Q1 journals. In fact, type 2 and 3 diseases are published in Q1 journals slightly more often than type 1a and 1b.

Figure 6b shows two indicators of citation impact across disease types. In this case, we find that type 1a and 1b diseases receive more citations than type 1c and 2, which are cited more often than type 3. The differences in citation are substantial – type 1a diseases get an 18% boost while type 3 receive 16% less citations than expected. Thus, the observations on JIF and citations offer different insights – diseases relevant for LMICs are equally accepted in high impact journals, but they receive less citations.

**Fig. 7:**
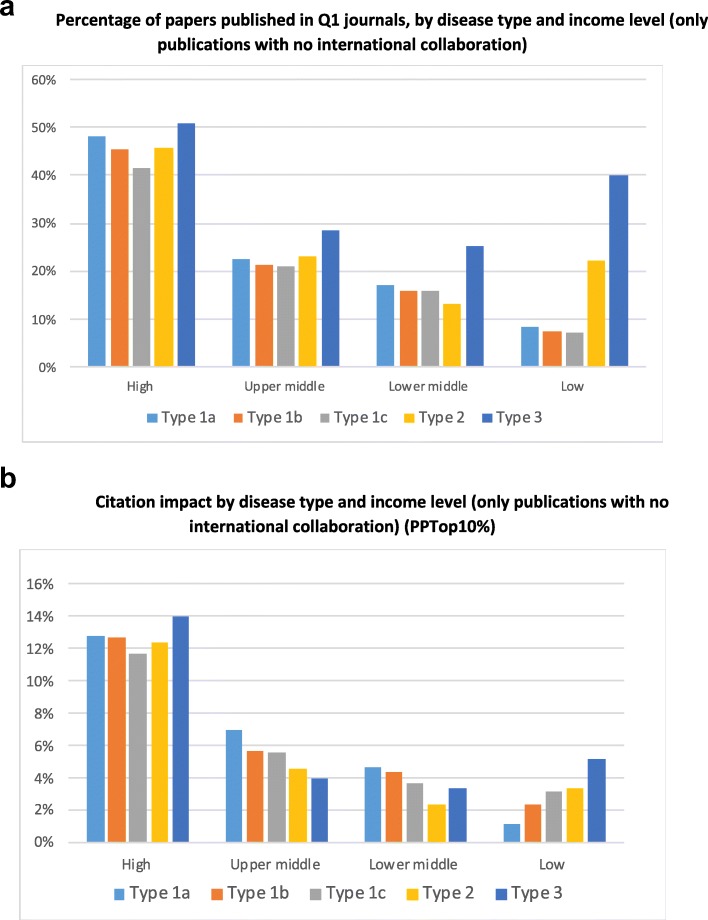
**a** Percentage of papers published in Q1 journals, by disease type and income level (only publications with no international collaboration). **b** Citation impact by disease type and income level (only publications with no international collaboration) *PPTop10%* Percentage of publications among the top 10% most cited

Next, we examined the distribution of Q1 and citations by country income level. In doing so, we found different patterns for publications produced with and without international partners. For LMICs, international publications have much higher JIF and citation rates than those without international partners. Since international collaborations account for about 20% of publications in HICs and MICs, but 75% of LICs (Fig. S4 in Additional file 2), this results in the artefact that LICs show higher citation rates than MICs. Therefore, for the sake of understanding the local capabilities to publish in prestigious journals and be cited, it is more informative to use publications produced by domestic research organisations only (Fig. 7a,b).

Figure 7a shows the percentage of publications in Q1 produced exclusively by domestic organisations across income classes. The percentage is rather similar in all disease types within each of the income levels, except for type 3, which has a higher proportion of publications in Q1. In the case of LoMICs and LICs, this proportion of Q1 is much higher, suggesting that they have a significantly higher expertise in type 3. Therefore, this observation questions the hypothesis that publishing in high impact journals is more difficult on diseases of LMICs.

Yet, when we turn to the citation impact, we see very different patterns at each income level (Fig. 7b). HICs achieve a similar citation impact in all disease types, with the highest for type 3. In LICs, citation impact increases steadily from type 1a to type 3, in accordance with its research specialisation. However, in UMICs and LoMICs, the opposite pattern is observed, i.e. citation rates decrease as one gets closer to type 3 diseases. This pattern may constitute a problem since it creates an incentive that does not fit with the needs suggested by the disease burden profiles in Fig. 3a. This possibility is particularly likely since many UMICs have individual evaluation or incentive schemes based on rather mechanistic bibliometric criteria, although often based on JIF quartiles rather than citations [28].

## Discussion

In this article, we have analysed patterns of disease burden in DALYs against research efforts as shown by publications across four country income levels and five disease types related to degree of burden by high/low income level. The study updates and corroborates previous analyses on the gap between global health needs and research efforts [10]. We find that type 1a diseases, with higher burden in HICs, receive ten-fold more research attention than diseases (type 3) affecting only LICs (Fig. 2b). A new finding is that the diseases affecting HICs and LMICs equally, and UMICs relatively more (types 1b and 1c), also receive two- to three-fold less attention.

We explored some of the key potential factors driving these imbalances of attention. First, we observe that health research is highly concentrated (80%) in HICs. As a result, it tends to focus on diseases more prevalent in these countries [5]. Even if efforts are made by some HICs to contribute to global health, these are insufficient to compensate for inequalities in R&D expenditure across countries. However, in the case of UMICs, we observe some problematic deviations from the expected patterns. Although UMICs contribute above expectation to the study of type 3 diseases, they also produce many more papers (1.5-fold more) than expected according to their own health needs on type 1a diseases (Fig. 4c) and less than expected (some 17% and 27% less) on type 1b and type 1c diseases. This observation suggests that, in UMICs, there might exist incentives, either in terms of funding or academic rewards, for researchers to publish in type 1a diseases.

Second, we observe that research conducted by pharma is also skewed towards type 1a and 1b diseases. The patterns of disease focus found for research published or funded by 23 big pharmaceutical companies are surprisingly similar to those found in public research. This suggests a strong coupling between private and public R&D in biomedical research.

Third, we do not find diseases more prevalent in LoMICs and LICs (types 2 and 3) to be underrepresented in the most prestigious journals of disciplines (in the top quartile of journal impact factor) – against the expectations of the literature studying marginalisation in mainstream journals of the topics specifically relevant to developing countries [31, 34, 46]. On the contrary, UMICs publish a bit more in top journals on types 2 and 3, while LICs publish much more. Given this pattern, LICs are found to have more citations in type 2 and 3 diseases, even when considering only domestic publications (Fig. 5b). Nevertheless, publications of types 1a and 1b have more citations than those of types 2 and 3 in aggregate (Fig. 4b). Importantly, this is also true in the domestic publications of MICs. These results are surprising – on average, in countries like China, India or Latin America, researchers may gain more citation impact if they publish in diseases with the highest burden in the global north. Those researchers in MICs receive more recognition when studying diseases that are not, in relative terms, in the top health priorities of their own context. This finding deserves further careful analysis at more fine-grained levels of aggregation to check its robustness.

The two main new findings of this paper concern the publication and citation impact of MICs. While there is a large volume of literature on poverty-related and neglected diseases [10], the analysis is generally focused on diseases affecting LICs. We are not aware of previous studies showing that diseases type 1b and 1c, which have a similar or slightly lower burden in HICs and LMICs, are relatively understudied. UMICs have now become a sizeable part of global science, accounting for 17% of disease-related publications worldwide. We have shown, first, that their research is partly focused (unlike that of LICs) towards diseases of HICs (type 1a). Second, we have found that, in UMICs, researchers may gain more citations by studying type 1a and 1b diseases, which may partially explain this deviation. This finding, which is still very speculative, would lend some credibility to the hypothesis that evaluation systems based in MICs divert attention away from their national contexts towards those of HICs. However, we also report that type 2 and 3 diseases appear as equally likely as type 1 to appear in prestigious journals and, in fact, formal incentive and evaluation programmes are more based on journal prestige than on citations received.

### Limitations and further research

This is an exploratory analysis with a variety of limitations that will benefit from further research. In the first place, DALYs are only one of the various possible forms to estimate disease burden – estimates based on other assumptions or understandings of health needs or well-being might yield different results [17, 36–38, 47]. Second, our bibliometric data is based on a database (Web of Science), which has been shown to underrepresent research LMICs [48, 49], and our search strategy is based on MeSH terms, which may overlook some non-biomedical publications. Third, the simple classifications of countries and diseases used are helpful in providing general patterns, but these general patterns should not be used to infer conclusions for individual countries given the risk of ecological fallacy. More fine-grained analyses are necessary, in particular given that large countries such as the United States (in HICs), China (in UMICs) and India (in LoMICs), strongly shape the results of their income level categories. Finally, we should keep in mind that, in order to improve the impact of research in global health, it is also necessary not only to address the relevant diseases, but also to improve other research strategies or approaches [1, 19]. In particular, it may be important to provide further support studies for implementation science or health systems research [11].

## Conclusions

In this study, we have corroborated that the relative imbalance of global health research to diseases most prevalent in poor countries can be mainly explained by the concentration of research in rich countries, where funding and incentives are likely to be driven by national rather than global needs [5]. We have also shed some light on two further mechanisms contributing to the lack of balance across health needs [20]. First, we have shown pharmaceutical companies focus on diseases with more burden in HICs (and thus larger market demand), which is likely to incentivise public researchers funded by industry to direct their research towards these diseases. Additionally, we have found that researchers in MICs receive more citations when conducting research on diseases more prevalent in HICs. This may constitute another incentive to publish in these areas, at the expense of other diseases more prevalent in their contexts.

Given that mainstream institutional structures tend to steer research on diseases more prevalent in HICs, policy initiatives will be needed for increasing research on diseases that are relatively unattended. First, as indicated by many previous studies, policies should encourage increasing the attention given to poverty-related and neglected diseases and a “*greater focus on actual knowledge exchange and transfer between LMICs and HICs* ( …)*, leading to strengthening of research capacity in the former*” [11]. Second, public and charity research funders should be aware of the potential influence of the pharmaceutical industry on public research system and balance their research portfolios in accordance to perceived needs and opportunities [50]. Third, it provides some (though not conclusive) evidence that, in MICs, notions of academic prestige based on citations might be a disincentive to work in diseases relevant to their context. This finding feeds into the discussion on the (perverse) effects of scientific reward systems in these countries, and the debate on introducing assessment criteria that directly support impact in health outcomes [31, 51].

## Supplementary information

**Additional file 1.** Detailed methodology.

**Additional file 2.** Data used in figures and tables, including supplementary material.

**Additional file 3.** Correspondence Table ICD-10 – MeSH.

## Data Availability

Data used in figures and tables is available in Additional file 2.

## Abbreviations

DALYs: disability-adjusted life years
HICs: high-income countries
ICD-10: International Classification of Diseases, 10th revision
IF: impact factor
JIF: Journal Impact Factor
LICs: low-income countries
LMICs: low- and middle-income countries
LoMICs: lower–middle-income countries
MeSH: Medical Subject Heading
Q1: Quartile 1 (i.e. 25% of journals with the highest Impact Factor in a given subject category)
R&D: research and development
UMICs: upper–middle-income countries
WoS: Web of Science

## Footnotes

1 Hagenaars et al. [7] propose to compute the disease burden based on its value in the quartile of countries where it is the lowest, since this value signals the disease burden according to the best available knowledge applied.

2 In other words, this indicator is the percentage of publications in journals that belong to the 25% of journals with the highest JIF within their Web of Science Subject Category, in the year when the article was published.
