# Supplementary material for: Exploring why global health needs are unmet by research efforts: the potential influences of geography, industry and publication incentives
Source: Health Res Policy Syst. 2020 May 15;18:47. doi: 10.1186/s12961-020-00560-6 (PMC7227286; doi:10.1186/s12961-020-00560-6)
Supplement: Supplementary file 1 — Additional file 1. Detailed methodology. [file 12961_2020_560_MOESM1_ESM.docx]

**Supporting Information for:**

**Exploring why global health needs are unmet by research efforts: the potential influences of geography, industry, and publication incentives**

Yegros-Yegros, A.; van de Klippe, W.; Abad Garcia, M.F. and Rafols, I.

**S1. Data and methods**

**S1.1 Estimates of disease burden**

We obtained burden of disease estimates from the World Health Organisation (WHO). The WHO provides regularly Global Health Estimates regarding mortality and loss of health, due to diseases and injuries. We downloaded the statistics on disease burden released in 2017, which provided estimates for the years 2000, 2005, 2010 and 2015 [1]. For this study we only used the estimates corresponding to the year 2010.

The various diseases are defined by the WHO following the International Classification of Diseases (ICD-10), according to a hierarchical structure. Each of the three most generic groups of diseases in this classification (i.e. *I. communicable, maternal, perinatal and nutritional conditions; II. Noncommunicable diseases and III. Injuries*), contain various sub-groups of diseases as well as specific diseases, which may be positioned at different levels in the hierarchy. For example, ‘*Drug use disorders*’ is a sub-group of diseases which include specific diseases such as ’*Opioid use disorders*’ or ‘*Cocaine drug disorders*’, among others. In turn, the sub-group ‘Drug use disorders’ is part of a more generic sub-group of diseases ‘Mental and substance use disorders’, within the ‘Noncommunicable diseases’. Within the same level of the hierarchy, all the categories are mutually exclusive, therefore there is no overlap in the burden estimates corresponding to different diseases in the same level of the hierarchy.

In this study we have considered the 134 most specific diseases in the hierarchy included in the groups *I. communicable, maternal, perinatal and nutritional conditions* and *II. Noncommunicable diseases*. In other words, we have excluded from the study both, any sub-group of diseases included in these two generic groups as well as the whole group of diseases ‘*III. Injuries’.*

The WHO estimates burden of disease according to different indicators, including Years Lived with Disability (YLD), Years of Life Lost and Disability Adjusted Life Year (DALYs). We have used DALYs as a measure of disease burden, as it combines both the number of years lost due to premature death and the number of years lived with ill-health or disability. DALYs results from summing up YLL and YLD.

DALYs are not free of limitations, previous studies have described in detail problems and limitations of this particular measure of disease burden [2–5]. The WHO itself also reflects on some challenges and limitations regarding the compilation of information to compute these estimates. For instance, it is acknowledged that ‘*large uncertainty ranges for some causes and some regions* [but still] *they provide useful on the relative importance of different causes of death and disability, and on regional patterns and inequalities*’ [1]. The WHO also warns that ‘*the data gaps and limitations in high-mortality regions reinforces the need for caution when interpreting global comparative burden of disease assessments*’ [1]. Even considering all these limitations, DALYs are probably the most established proxy of disease burden.

DALYs were estimated for 183 WHO Member States, those with populations greater than 90,000 in 2015. This threshold excluded 11 WHO Member States: Andorra, Cook Islands, Dominica, Marshall Islands, Monaco, Nauru, Niue, Palau, Saint Kitts and Nevis, San Marino, and Tuvalu. Although DALYs are also provided disaggregated by sex and various age groups, we have used DALYS for the whole population, without distinguishing by sex or age.

Further details on the methodology followed by the WHO in the compilation of burden of disease for the years 2000, 2005, 2010 and 2015, can be found in the ‘WHO methods and data sources for global burden of disease estimates 2000-2015’ [1].

**S1.2 Publication data**

We have considered the number of publications as a proxy of the research effort made around the various diseases, which may result of any type of funding, either institutional (i.e. block grants) and external funding (i.e. project funding). Other measures are also often used in this context, such as the investment made in each disease according to project budgets. However, the latter seems more accurate as a proxy of financial efforts rather than research efforts, as it fails to capture research carried out by academic researchers or medical doctors without specific funding. In any case, exploratory studies suggest that both proxies are highly correlated (Ciarli & Ràfols, 2019, p. 955). We acknowledge that the number of total research articles is not a perfect indicator, but it can be considered a reasonable indicator of health research [7]

We collected scientific publications through PubMed, which provides access to more than 30 million scientific publications included in the MEDLINE database. MEDLINE is the bibliographic database on biomedical literature produced by the U.S. National Library of Medicine in the U.S (NLM). The NLM uses a controlled vocabulary (Medical Subject Headings - MeSH) to describe the content of the scientific articles included in the database, index them and support the search of information.

In order to enable the comparison between the burden of disease estimates and the number of publications corresponding to a specific disease, we identified the MeSH terms that best describe each of the 134 diseases included in our study (MeSH version 2018). To create this correspondence between MeSH terms (linked to publications in MEDLINE) and ICD-10 (burden of disease, from WHO) we relied on several resources, such as the Unified Medical Language System (UMLS), a tool produced by the NLM which provides information on the relationship between concepts from different classifications, so that with this tool we were able to identify the MeSH terms corresponding to a number of ICD-10 codes.

Given that the UMLS did not provide MeSH terms for each of the ICD-10 codes corresponding to the 134 diseases, we also used complementary strategies. We also used Wikipedia to collect ICD-10 – MeSH candidate pairs to be included in our correspondence table. Wikipedia often includes in the article of the disease information on the codes from various classifications corresponding to the disease, including ICD-10 and MeSH, among others.

For remaining ICD-10 codes corresponding to our 134 for which we were not able to identify relevant MeSH terms through the previous strategies, we directly assigned the most adequate MeSH term by searching directly the MeSH database.

The assignment of MeSH terms to ICD-10 codes was reviewed by one of the authors (Professor Abad-García) who is a medical doctor, with an extensive experience working with biomedical and disease classifications.

To retrieve publications related to the various diseases, we included in the search not only the MeSH terms we previously assigned to the various ICD-10 codes, but also any specific terms beneath them, according to the MeSH structure. This procedure is the so called ‘automatic explosion’, and it is automatically performed in PubMed. This approach allowed us to assign, for instance, to the disease “malaria” in the classification of the WHO, publications with the MeSH term “Blackwater Fever” even if the MeSH term “malaria” was not assigned them.

All MeSH terms are included in one or more of its 16 main categories (from *A- Anatomy* to *Z- Geographicals*), according to a hierarchical structure. A given MeSH term might be classified as specific term of two or more generic terms, within the same main branch or in different branches. Therefore, unlike the non-overlapping criteria followed by the WHO to assign the burden of disease across categories, the MeSH tree structure might lead to duplicate assignments of publications to diseases.

In order to be consistent with the methodology followed by the WHO, we checked those MeSH terms that, due to the ‘automatic explosion’ procedure and the nature of the MeSH tree structure, ended up assigned to more than one of the 134 diseases as considered by the WHO. While checking these possible MeSH duplicates, we kept a given MeSH term assigned only to one of the WHO disease and removing all the unintended assignments, following the same criteria as the WHO.

This can be illustrated with the WHO disease “Ovary cancer” (ICD-10 = C56), for which we assigned as primary MeSH term “Ovarian Neoplasms”. However, the ‘automatic explosion’ automatically assigned the MeSH “Ovarian Neoplasms” also to the WHO disease “Gynecological diseases”. This happens because according to the MeSH tree structure, “Ovarian Neoplasms” is a specific term of “Genital Diseases, Female”. In this case, we removed the MeSH “Ovarian neoplasms” from the WHO disease “Genital Diseases, Female” and we only used this MeSH term to assign publications to the WHO disease “Ovarian Neoplasms”.

**Table S1. Example of multiple assignation in the**

| **WHO disease** | **MeSH assigned** | **Specific MeSH** |
| --- | --- | --- |
| Gynecological diseases | Genital Diseases, Female | Ovarian Neoplasms (removed) |
| Ovary cancer | Ovarian Neoplasms | - |

However, publications might still be assigned to two different diseases, but only when they have been indexed with two or more non-overlapping MeSH terms linked each of them to different WHO diseases.

When necessary, we used qualifiers in combination with MeSH terms. For instance, we used the qualifier ‘congenital’ together with some MeSH assigned to some congenital conditions.

The number of publications produced for each disease was computed using fractional counting. This means that if a given publication is classified in two different WHO diseases, we counted half publications for each disease. For instance, a publication classified on ‘Acute hepatitis B’ and also ‘liver cancer’ is counted as 0.5 for each disease.

Both, the collection of publications related to each disease and also in the mapping of MeSH and WHO classifications are not free of limitations. MeSH is a comprehensive vocabulary, but “*it should not be regarded as representing an “authoritative subject classification system but rather as arrangements of descriptors for the guidance and convenience of persons who are assigning subject headings to documents or are searching for literature. The trees are not an exhaustive classification of the subject matter but contain only those terms that have been selected for inclusion in this thesaurus*”^[[1]](#footnote-1)^. Thus, the possibilities of finding appropriate terms for each disease partially depend on the availability of MeSH terms in the database.

We also rely on the assignment of MeSH terms to publications done by human indexers, and therefore not exempt of problems (e.g. Minguet et al, 2015). Also, some records in the database do not have MeSH terms assigned, and therefore we could not retrieve these records when using MeSH terms to search the database. There are for instance publications that might be relevant for a particular disease, as for instance malaria (e.g. pmid 21151372 or pmid 21476815), but there are not MeSH terms assigned to these records. We did not explore in detail how many records potentially relevant for the various diseases covered by our study do not carry MeSH terms, but these examples illustrate that this aspect might affect the collection of publications. Sometimes the absence of MeSH terms might be related to the delay in indexing records by the NLM [8,9]. However given the time period covered in our study (2010-2014) it is not clear whether the absence of MeSH terms in this period is only related to the delay on indexing or if some other factors are in play.

Regarding the mapping exercise, in some cases the hierarchical structures in ICD-10 and MeSH seem to follow different criteria, for instance, the “Plummer-Vinson syndrome” is classified under D50.1 in the ICD-10, therefore under “Iron deficiency anemia” (D50). However, the MeSH term “Anemia, Iron-Deficiency” has not “Plummer-Vinson syndrome” as specific term. The MeSH classifies the term “Plummer-Vinson syndrome” under “Esophageal Motility Disorders”, and thus considers this syndrome as a digestive system disease. These differences in the classification implies that seemingly equivalent terms (the ICD-10 “Iron deficiency anemia” and the MeSH “Anemia, Iron-Deficiency”) differ in terms of their scope. We tried to align both classifications as much as possible, but given the complexity of the task we cannot ensure that our concordance table fully accomplished this alignment, and thus is open for further improvements.

We acknowledge that all these problems may impact the comprehensiveness and accuracy in the identification and assignment of publications to diseases. We do not claim that our data collection is complete, accurate and free of limitations, but we do consider that this approach provides an acceptable estimate of the volume of scientific publications produced around the selected diseases. Also important, our methodology can be fully replicated and others may improve it in the future.

After collecting all publications from PubMed, we linked all these records to the Clarivate Analytics’ CWTS in-house version of the Web of Science (WoS). We relied on WoS to collect complete information on the affiliation of the authors and to collect the number of citations received by publications. We only considered in our study articles and reviews, as we consider these types of documents reflect best the research efforts compared to other type of documents included in the database, such as editorial material, book reviews and others. We considered publications produced in the period 2010-2014, to ensure that there was sufficient time for these publications to accumulate citations.

As shown in Figure S1, we estimate that the 134 diseases covered in our study amount to 60% of all publications on diseases^[[2]](#footnote-2)^ (around 13% of total WoS, about 900k papers over 5 years). It is about one third of all health-related research in WoS^[[3]](#footnote-3)^, which makes about 42% of WoS as a whole. Health research funders often fund research which is not listed [10].

**Figure S1 Percentage of articles and reviews (2010-2014) relative to all WoS devoted to health related research, diseases and the 134 diseases covered in the study**

**S1.3 Publications produced and funded by big pharma**

In order to analyse the priorities of big pharma across disease types, we have considered 23 large pharmaceutical companies. Thus, instead of considering an exhaustive list of companies active in the pharma sector, we focus on a small group of companies, representing the largest companies in this sector.

Most of these companies are among the top 100 companies worldwide in terms of R&D investment. According to EU Industrial R&D Investment Scoreboard, produced by the EC’s Joint Research Centre in Seville (Spain). These 23 companies together invested more than 97 billion euros in R&D in 2016 and achieved sales for more than 600 billion in the same year.

In terms of the geographic distribution of headquarters, 11 of the selected companies are located in the US, 9 in Europe, 2 in Japan and one company in Israel.

For the collection of their scientific publications, we considered all subsidiaries as well as acquisitions. To define and delineate these companies we relied on Moody’s Orbis, which is probably the largest commercial database providing corporate information, supplemented with other written sources (mainly Wikipedia and [11]).

Past publications produced by any acquisition or subsidiary have been considered. We made two exceptions, the acquisition of Monsanto by Bayer AG as well as Eli Lilly’s Elanco have not been considered in the analyses as their main focus is other than pharmaceuticals.

The identification of research publications by pharma companies was conducted in August 2017, following the existing corporate structure at that moment in time.

We retrieved two sets of publications related to these large pharmaceutical companies. On one hand, we retrieved publications in which the pharma company is listed as affiliation of at least one of the authors of the publications. On the other hand, we identified publications in which authors acknowledge funding from there pharma companies. These two sets of publications are not necessarily mutually exclusive, as some publications (co-)authored by employees of the pharmaceutical company may also acknowledge having received funding from the company where they work.

The acknowledgements, as collected by WoS, often include statements reflecting potential conflicts of interest, and this information is frequently mixed in the database with statements referring to actual funding acknowledgements [12]. To make sure that we include in the analysis only publications (partially) funded by pharma companies, we excluded from the analysis any publication where the pharma company is only mentioned next to an expression typically used in the context of conflicts of interest.

**S1.4 Country classification**

We have considered in the study the 183 countries for which the WHO provided disease burden estimates. Publications are assigned to countries of affiliations of the authors, applying fractional counting. If a publication produced by authors from four different countries, we assigned 0.25 publications to each country.

Countries have also been grouped into 4 categories according to their income level in the year 2010, following the World Bank’s historical classification of countries by income^[[4]](#footnote-4)^: High Income (HICs), Upper-middle income (UMICs), Lower-middle income (LoMICs) and Low income (LICs).

The distribution of publications by country in these categories is highly skewed, most of the scientific production is concentrated in a low number of countries. The US generated 35% of publications of HICs), China produced half of the publications in the UMICs (50%), while India produced 60% of all pubs by LoMICs. These countries strongly shape the results of their category. The distribution is similar in the LICs, but here the countries with higher number of publications account for a lower percentage of publications in the category (e.g. each of the top producers like Kenya or Uganda represent around 15% of publications).

**S1.5 Disease classification**

The 134 diseases considered in our study are also analysed at an aggregate level. inspired by the framework developed by the WHO [13], that groups specific diseases into types of diseases to provide a method to inform debates on the scope of any R&D monitoring activities [14].

Prior to the categorisation of diseases, we applied the same calculations as described by the WHO. We computed, for each specific disease, a ratio between the aggregated DALYs per 100,000 population in LICs/LoMICs/UMICs and the aggregated DALYS per 100,000 population in HICs. A ratio of 1 would indicate the disease is found in equal measure in all countries. A ratio lower than 1 would indicate that the disease affects relatively more to HICs, while if the ration is higher than 1 the disease would be affecting relatively more to Low/middle income countries.

Originally the WHO suggested three groups of diseases (disease types 1, 2 and 3), however we created five categories with to the following ratio ranges (see also Table 1 in main text):

- Type 1a < 0.75
- Type 1b 0.75 ≤ x < 1.25
- Type 1c 1.25 ≤ x < 3.00
- Type 2 3.00 ≤ x < 35.0
- Type 3 ≥ 35.0

The difference between our categories and the original proposed by WHO is that we have further split the original type 1 group of diseases into three subgroups (a, b and c). Similarly, others have also used five categories of diseases, but creating the categorisation according to slightly different ratio ranges [15].

Similarly to the distribution of publications by country within the various income level groups, some diseases in each disease type concentrate a higher number of publications. In type 1a diseases, breast cancer accounts for 13.4% of publications, in type 1b diabetes mellitus represents roughly 20% of publications, in type 1c 22.4% of publications related to skin diseases, in type 2 maternal conditions concentrates 33% of all publications, while in type 3 Tuberculosis represents slightly over 26% of all publications.

**S1.6 Citation impact**

We have sued two different indicators based on citation counts as proxies of the scientific impact of publications. One of the indicators is the Mean Normalised Citation Impact (MNCS), that measures the average citation impact of a set of publications. The second measure indicates the proportion of publications among the top 10% most cited worldwide PP(Top 10%).

Both indicators are normalised by scientific field and publication year. This means that the publications we analyse in our study are compared to publications worldwide published the same year and in the same scientific field. To normalise by field, we relied on the WoS Subject categories. It is very important to perform this type of normalisations in to control for possible differences in the citations received due to the time of publication of the different propensity in which scientific publications are cited in different scientific fields [16,17].

The first indicator, MNCS takes the actual number of citations received by each publication and then it is divided by the average number of citations received by all the publications published the same year in the same scientific field worldwide. Once these values have been computed for all individual publications, then they are aggregated to obtain the corresponding MNCS

The PP(Top 10%) indicates the proportion of publications produced by a given aggregate that, compared with other publications in the same scientific field and published in the same year, belong to the top 10% most frequently cited. In order to obtain this indicator, we first give a citation score of 1 to individual publications that belong to the top 10% most cited publications worldwide, while the score is 0 otherwise. The average citation score of a given aggregate yields to final score of the PP(Top 10%).

We also collected the Impact Factor (IF) of the journals where the articles included in our analysis where published. This information was extracted from the Clarivate Analytics’s Journal Citation Reports (JCR). It reflects the average frequency with which articles published in a given journal are cited in a given period of time. The IF of a journal in a given year is computed as a ratio, considering as numerator the total number of citations in that particular year to articles published in that journal during the two precedent years and, as denominator, the number of articles published in these two precedent years^[[5]](#footnote-5)^,^[[6]](#footnote-6)^.

Based on all values of the IF, we were able to determine the quartile to which the journal belongs. We followed the same procedure as in the JCR^[[7]](#footnote-7)^ to obtain these quartiles, by dividing the journal rank (journals are ordered by IF from higher to lower) (X), by the number of journals in the number of journals classified in the same subject category (Y). This division results in a value (Z = X / Y) that we use to distribute journals in quartiles (Q):

- Q1: 0.0 < Z ≤ 0.25
- Q2: 0.25 < Z ≤ 0.5
- Q3: 0.5 < Z ≤ 0.75
- Q4: 0.75 < Z

Thus, Q1 contains the 25% of the journals from a given subject category with the highest IF, while the 25% of journals from the same subject category with the lowest IF are in Q4. Journals might be classified in more than one subject category and, as a result of these calculations, the same journal may be classified in different quartiles. When this happens we have considered the best quartile, for instance for a journal in Q2 and Q1 we have considered the journal to belong to Q1.

**S2. Further results**

This section provides empirical results that are not central to the main argument of the article, but that complement and help interpret the findings.

**S2.1 Distribution of burden and efforts across income levels for a given disease type**

Figure S2 shows the distribution of disease burden for each disease types across income levels. We can observe that HICs (in blue) contribute almost 30% of the burden for type 1a, but smaller proportions for type 1b, 1c and 2. On the other hand, most of the burden for type 2 and 3 correspond to LoMICs and LICs. Figure S3 shows that in type 3 diseases the contribution of non-HICs covers a higher proportion of the publications.

**Figure S2 Distribution of DALYs across income levels for a given disease type**

**Figure S3. Distribution of publications across income levels for a given disease type**

**S2.2 International collaboration patterns**

Figure S4 shows that LICs are highly dependent on international collaborations for publishing in journals classified in WoS. Similarly, international collaborations constitute about 20% of type 1a, 1b and 1c diseases, 25% of type 2 diseases, but 40% of type 3 diseases (shown in Fig. S5).

**Figure S4. International collaboration by income level**

**Figure S5. International collaboration by disease type**

**S2.3 Journal prestige and citation patterns with international collaboration**

In the case of international collaborations, shown in Figure S6, there is a substantial decrease in the percentage of Q1 publications in type 1a, 1b and 1a, from 60% in HICs to 40% in LICs, but this decrease is much smaller than that observed in Figure 5a, from about 45% to less than 10%. Similarly Figure S7 shows that citation impact for different income groups are quite similar – which is not the case without international collaboration. In summary, Figures S7 and S8 illustrate the strong effect that international collaboration in raising visibility and citation impact of publications.

**Figure S6. Percentage of papers published in Q1 journals, by disease type and income level (with international collaboration)**

**Figure S7. Citation Impact (PP(top10%), by disease type and income level (with international collaboration)**

A potential confounding factor is that LMICs tend to have lower coverage in databases sucha as WoS [18,19]. Indeed, using as a proxy the indicator of ‘internal coverage’ (i.e. the percentage of references from the publications included in the WoS), coverage of diseases in the WoS can be shown to slightly higher for type 1 diseases – which means that there more papers of type 2 and 3 published in ‘lower prestige’ are not captured in our data. However, these differences are relatively small, as shown in Figure S9. Type 1a diseases have 91% internal coverage, whereas type 2 and 3 have 86% and 84%, respectively. Similarly, HICs have a coverage of 90%, MICs of 89%, LoMICs of 84% and LICs of 79%. Correcting for these differences in the order of 5%-10%, the percentage of publications across disease types would be even more similar across disease types.

**Figure S8. Internal coverage (percentage of references included in the database)**

**References**

1. WHO. Disease, injury and causes of death country, regional and global estimates, 2000–2015 [Internet]. 2017 [cited 2019 Oct 28]. Available from: https://www.who.int/healthinfo/global_burden_disease/estimates_country_2000_2015/en/

2. AbouZahr C, Boerma T, Hogan D. Global estimates of country health indicators: useful, unnecessary, inevitable? Glob Health Action. 2017 Dec;10(sup1):1290370.

3. Anand S, Hanson K. Disability-adjusted life years: a critical review. J Health Econ. 1997 Dec;16(6):685–702.

4. Arnesen T, Nord E. The value of DALY life: problems with ethics and validity of disability adjusted life years. BMJ. 1999 Nov 27;319(7222):1423–5.

5. Parks R. The Rise, Critique and Persistence of the DALY in Global Health. J Glob Health [Internet]. 2014 [cited 2019 Oct 28]; Available from: //www.ghjournal.org/the-rise-critique-and-persistence-of-the-daly-in-global-health/

6. Ciarli T, Ràfols I. The relation between research priorities and societal demands: The case of rice. Res Policy. 2019 May 1;48(4):949–67.

7. Evans JA, Shim J-M, Ioannidis JPA. Attention to local health burden and the global disparity of health research. PloS One. 2014;9(4):e90147.

8. Irwin AN, Rackham D. Comparison of the time-to-indexing in PubMed between biomedical journals according to impact factor, discipline, and focus. Res Soc Adm Pharm RSAP. 2017 Apr;13(2):389–93.

9. Rodriguez RW. Delay in indexing articles published in major pharmacy practice journals. Am J Health-Syst Pharm AJHP Off J Am Soc Health-Syst Pharm. 2014 Feb 15;71(4):321–4.

10. Grassano N, Rotolo D, Hutton J, Lang F, Hopkins MM. Funding Data from Publication Acknowledgments: Coverage, Uses, and Limitations. J Assoc Inf Sci Technol. 2017;68(4):999–1017.

11. Rafols I, Hopkins MM, Hoekman J, Siepel J, O’Hare A, Perianes-Rodríguez A, et al. Big Pharma, little science?: A bibliometric perspective on Big Pharma’s R&D decline. Technol Forecast Soc Change. 2014 Jan 1;81:22–38.

12. Lewison G, Sullivan R. Conflicts of interest statements on biomedical papers. Scientometrics. 2015 Mar 1;102(3):2151–9.

13. WHO. Defining disease types I, II and III [Internet]. 2012. Available from: https://www.who.int/phi/3-background_cewg_agenda_item5_disease_types_final.pdf

14. Røttingen J-A, Regmi S, Eide M, Young AJ, Viergever RF, Ardal C, et al. Mapping of available health research and development data: what’s there, what’s missing, and what role is there for a global observatory? Lancet Lond Engl. 2013 Oct 12;382(9900):1286–307.

15. von Philipsborn P, Steinbeis F, Bender ME, Regmi S, Tinnemann P. Poverty-related and neglected diseases - an economic and epidemiological analysis of poverty relatedness and neglect in research and development. Glob Health Action. 2015;8:25818.

16. Waltman L, van Eck NJ. Field-normalized citation impact indicators and the choice of an appropriate counting method. J Informetr. 2015 Oct 1;9(4):872–94.

17. Waltman L, van Eck NJ, van Leeuwen TN, Visser MS, van Raan AFJ. Towards a new crown indicator: Some theoretical considerations. J Informetr. 2011 Jan 1;5(1):37–47.

18. Chavarro D. Universalism and particularism: explaining the emergence and development of regional journal indexing systems [Internet]. [Brighton]: SPRU - University of Sussex; 2017 [cited 2019 Oct 28]. Available from: http://sro.sussex.ac.uk/id/eprint/66409/

19. Mongeon P, Paul-Hus A. The journal coverage of Web of Science and Scopus: a comparative analysis. Scientometrics. 2016 Jan 1;106(1):213–28.

1. <https://www.nlm.nih.gov/mesh/intro_trees.html> [↑](#footnote-ref-1)
2. We estimated the total amount of publications dealing with specific diseases by looking at the number of publications with at least one MeSH term from the branch ‘C’ [↑](#footnote-ref-2)
3. This estimate is based on the number of publications published in journals classified in WoS Subject categories relevant in biomedical research. The list of these 84 categories can be found in the supplementary material. [↑](#footnote-ref-3)
4. <https://datahelpdesk.worldbank.org/knowledgebase/articles/906519-world-bank-country-and-lending-groups> [↑](#footnote-ref-4)
5. https://clarivate.com/webofsciencegroup/essays/impact-factor/ [↑](#footnote-ref-5)
6. While the numerator includes all citations to any article published by the journal, the denominator only the so called ‘citable items’, which refer to articles and reviews. Therefore, any other type of publication such as editorial material, letters, etc. are excluded from the denominator. [↑](#footnote-ref-6)
7. <http://help.incites.clarivate.com/inCites2Live/indicatorsGroup/aboutHandbook/usingCitationIndicatorsWisely/jifQuartile.html> [↑](#footnote-ref-7)
